# Pulmonary T2* quantification of fetal lung status in congenital diaphragmatic hernia: future alternative to ultrasound?

**DOI:** 10.1038/s41390-025-04241-4

**Published:** 2025-06-24

**Authors:** Anna Foth, David G. Tingay

**Affiliations:** 1 Paediatric Intensive Care Unit, Children’s Health Ireland at Crumlin, Dublin, Ireland; 2 Neonatal Research, Murdoch Children’s Research Institute, Parkville, Australia VIC; 3 Department of Paediatrics, University of Melbourne, Melbourne, VIC Australia; 4 Department of Critical Care, University of Melbourne, Melbourne, VIC Australia

Congenital diaphragmatic hernia (CDH) is characterized by herniation of abdominal contents into the thoracic cavity in fetal life. This impairs lung and heart growth and results in pulmonary hypoplasia, pulmonary vascular disease and left ventricular dysfunction, the magnitude of each impacting the severity of neonatal presentation and response to treatment.^1^ Neonatal management is difficult and limited to supportive care after surgical repair. Consequently, mortality and morbidity remain high compared to other neonatal cardiorespiratory disorders despite significant advances in critical care support. It has long been recognized that the magnitude of fetal pulmonary hypoplasia is a predictor of neonatal outcomes.^2^ To date the mainstay of antenatal assessment has been ultrasound (USS), with observed to expected lung head ratio (O/E LHR) the most often used, and validated, measure of pulmonary hypoplasia and postnatal prognostication.^3^ USS, and O/E LHR in particular, are not without limitations.^2^

There has been increasing interest in the use of fetal MRI (fMRI) as an alternative to USS in pregnancies with CDH.^4^ Thus, the study of Avena-Zampieri and co-workers in this edition of *Pediatric Research* is timely and of interest.^5^ Avena-Zampieri and co-workers aimed to report the potential of a new fMRI method (T2* relaxometry with motion correction) to assess lung volume in fetuses with CDH. In their retrospective case-control study they report lung volumes (total, ipsilateral and contralateral) using T2* relaxometry fMRI in 12 fetuses with CDH (9 left and 3 right CDH; 5 survivors), and compared these to 34 control fetuses without CDH (one excluded) who had fMRI for other reasons as part of other studies. The fMRI technique is provided in detail, critical was the use of an in-house reconstruction process (deformable slice to volume reconstruction; DSVR) that allowed for motion-correction to generate 3D data.^6^ fMRI that met image quality rules were then processed with a deep-learning algorithm to segment lung tissue from other structures and calculate lung volume.

Reassuringly, Avena-Zampieri and co-workers found that lung volumes were smaller in the CDH cohort compared to control; mean (95%CI) 16 (10,19) cc or 24 (4,41) ms (pulmonary T2*) lower than controls. Further ipsilateral lung volume was lower than contralateral. Within the control group, lung volumes and pulmonary T2* values correlated to gestational age, and thus suggest the technique could map fetal lung growth. Lung volume and pulmonary T2* values were relatively similar across fetal gestations in the CDH cohort. The reason for the timing of fMRI in each CDH case is unknown, which is important as gestation at fMRI was not standardized and appeared to be later in non-survivors. The authors reported that contralateral, but not ipsilateral, lung volume was lower than controls in those infants with CDH that did not survive. In contrast pulmonary T2* values in both lungs were lower in non-survivors. Consistent with the high mortality rate in the CDH cohort (58%), pulmonary hypertension was common, and 7 of the 9 infants who underwent early echocardiographic assessment had dysfunction of one or both ventricles. Neonates were treated at several different centers, although the authors pointed out that the protocols were similar. Additionally, three of the twelve children with CDH had a structural cardiac abnormality, accounting for nearly 42% of the non-survivors. This represents a significant confounding factor.

The main value of Avena-Zampieri and colleagues work is to demonstrate feasibility of a new technique. CDH is a rare condition, and infants often critically unwell, thus research in this population is difficult. Clinical practice has often been meaningfully influenced by small physiological studies.^7^ The authors should be congratulating in recruiting 12 families from a single center for complex fMRI imaging. Now that the principle of pulmonary T2* relaxometry has been demonstrated in CDH it will be interesting to see how the authors progress the concept, particularly whether its role is limited to research or can be applied in a broader clinical settings. If the authors aim to explore the latter, the recent use of a coordinated registry network to conduct a high-quality observational study on the role of left and right ventricle dysfunction serves as a model to follow.^1^

A recent priority setting partnership project identified 1) the best antenatal predictors of outcome in CDH and 2) whether antenatal interventions can improve outcomes as unmet research priorities in CDH (ranked #15 and #9, respectively).^8^ The TOTAL trial has further reinforced the importance of antenatal prognostication of CDH risk. The TOTAL trial demonstrated that fetal tracheal occlusion improved neonatal survival in fetuses with severe CDH.^9^ In moderate CDH the benefit was less clear,^10^ with utility of fetal intervention being heavily dependent on reliability of antenatal prognostication and anticipated local neonatal practices due to the maternal morbidity and increased risk of preterm birth.^10^ O/E LHR was the mainstay for categorizing severity,^3^ and 10 infants in this study had reported values between 18 and 75% suggesting a spectrum of neonatal risk. O/E LHR is not a panacea for prediction as fetal lung size is not an absolute proxy of function or fetal growth trajectory. Volumetric assessment of lung volume using fMRI has been suggested as a more accurate assessment of pulmonary risk but still confers the limitation of lung size being an inaccurate correlate of postnatal function.^4^ T2*relaxometry adds a measure of lung function to fMRI, holding appeal as a a fetal tool in CDH.^6^ Compared to USS, access to fMRI remains a barrier to clinical generalization. fMRI requires specialized personnel and complex post-processing techniques.

T2*relaxometry describes how the magnetization of tissue decays during imaging, and thus is a proxy of tissue inhomogeneity, as in any given tissue differences in function and structure will alter its magnetic properties. T2*relaxometry has been proposed as a tool to assess tissue perfusion/oxygenation (oxygenated and deoxygenated tissue will have different magnetic properties; the blood O2 level-dependent effect) in addition to simple anatomical assessment. As T2*relaxometry reflects magnetic decay it is measured in a unit of time (ms) rather than volume. T2* relaxation requires tissue perfusion to detect inhomogeneities. Importantly, the ability of T2* relaxation in the fetal lung, which is not actively engaging in gas exchange and has reduced pulmonary blood flow, to define lung function will be different from the postnatal lung. Fetal tissue T2* values also change with gestational age. CDH does alter fetal metabolic status, and causes hypertrophy of pulmonary arteries, vasoconstricted vascular smooth muscle cells, and reduced vascular branching.^7^ These vascular changes would be expected to impair pulmonary perfusion, providing a biological rationale to the reported lower mean T2* values compared to controls. This underscores the preliminary nature of the current study, but also the potential for T2* relaxometry to contribute to the assessment of fetal lung vasculature development and prognostication in CDH. It also reinforces the need for larger registry studies that can integrate multiple imaging modalities to provide a more comprehensive understanding of fetal lung development and function in CDH.

The conclusions regarding the ability of pulmonary T2* to prognosticate neonatal CDH outcomes should be interpreted with caution. In addition to being a small uncontrolled population, the side of hernia, magnitude of pulmonary hypoplasia size, and fetal age at imaging varied. The CDH cohort also had a high mortality (58%), and high rate of CHD (25%), with non-survivors imaged at a later gestation, when the difference between control group was the greatest. Minimizing selection biases will be important in determining the generalizability of fMRI T2* relaxometry to predict CDH outcomes, especially when comparing to the well-established and studied use of USS.

Recent data on embryology and postnatal development of neonates with CDH highlight the crucial role of vasculogenesis.^1,7^ Historically, CDH was considered primarily a disease of hypoplastic lungs. Recent data have shifted focus to the cardiopulmonary interaction, emphasizing the predominant role of hemodynamic and distinguishing different hemodynamic phenotypes.^1^ This suggests that precise management based on cardiac dysfunction phenotypes could improve survival rates and long-term outcomes independent of pulmonary hypoplasia.^1^ CDH is a complex condition with a potential embryopathy background; future fMRI studies and applications should also include assessment of cardiovascular development.

The use of a deep-learning tool to automate segmentation of fetal/neonatal lung tissue is an important advancement. Tissue segmentation tools are widely used in adult computerized tomography and MRI practice, but the use in neonates and fetuses has been more complex and often required some human oversight to calculate lung volume.^11^ The authors attempted to mitigate segmentation error, with a good agreement between experienced manual and deep-learning segmentation. Further work will be needed to better understand whether surrounding structures were avoided in segmentation (and miscalculation of volume) given the very different chest contents of the CDH and control groups. Hopefully, the authors will further develop and validate their tool into one that has broader antenatal and perinatal uses.

The study of Avena-Zampieri and co-workers introduces a fascinating technique to address an important clinical knowledge gap in those born with CDH. The addition of T2* relaxometry to fMRI may offer insight into the developmental state of the fetal CDH, with potential benefit compared to USS being the trajectory of the measure with gestation.
