# Correction: Pulmonary T2* quantification of fetal lung status in congenital diaphragmatic hernia: future alternative to ultrasound?

**DOI:** 10.1038/s41390-026-04923-7

**Published:** 2026-03-24

**Authors:** Anna Foth, David G. Tingay

**Affiliations:** 1 Paediatric Intensive Care Unit, Children’s Health Ireland at Crumlin, Dublin, Ireland; 2 Neonatal Research, Murdoch Children’s Research Institute, Parkville, VIC Australia; 3 Department of Paediatrics, University of Melbourne, Melbourne, VIC Australia; 4 Department of Critical Care, University of Melbourne, Melbourne, VIC Australia

Correction to: *Pediatric Research* https://doi.org/10.1038/s41390-025-04241-4, published online 24 June 2025

In this article, the author’s name Anna Foth was incorrectly written as Anna Forth. The original article has been corrected.

